# Patients’ Experiences Using Closed Incision Negative Pressure Wound Therapy Dressing After Infra-Inguinal Vascular Surgery

**DOI:** 10.1177/23743735221112595

**Published:** 2022-08-15

**Authors:** Johan Nyman, Stefan Acosta, Christina Monsen, Julien Hasselmann, Francis Rezk, Ann-Christine Andersson

**Affiliations:** 1 Department of Clinical Sciences, 59568 Lund University, Malmö, Sweden; 2 Vascular Center, Department of Cardiothoracic and Vascular Surgery, Skåne University Hospital, Sweden; 3 Department of Allied Health Professions, Skåne University Hospital, Sweden; 4 Department of Surgery, Unit of Vascular Surgery, Jönköping County, Jönköping, Sweden; 5 Department of Surgery, Skåne University Hospital, Unit of Vascular Surgery, Jönköping County, Jönköping, Sweden; 6 Jönköping Academy, 4161 Jönköping University, Jönköping, Sweden; 7 Jönköping, and Department of Care Science, Malmö University, Malmö, Sweden

**Keywords:** surgical site infection, incision, negative pressure wound therapy, patients’ experience, dressing, vascular surgery

## Abstract

The PICO™ dressing utilizes incisional negative pressure wound therapy in reducing surgical site infection after vascular surgery; however, no patient-reported investigations are available. The objective was to explore patientś experiences wearing the PICO™ dressing for 7 days. Nine men and 6 women were interviewed, and analysis was conducted using qualitative content analysis. The PICO™ dressing system was well accepted by most patients. Most prominent problems were fear of dropping the pump to the floor, lack of information, and initial feelings of uncertainty. Four patients who had the PICO™ and standard dressing in opposite groins simultaneously, preferred the PICO™ dressing.

## Introduction

A new technique for the prevention of surgical site infection is incisional negative pressure wound therapy (iNPWT). A wound dressing is placed upon the closed incision under sterile conditions, providing constant negative pressure of 80 mm Hg for 7 days. The dressing offers a tight seal of the wound, strengthens the sutures (1), and reduces edema (2).

**Figure 1.**
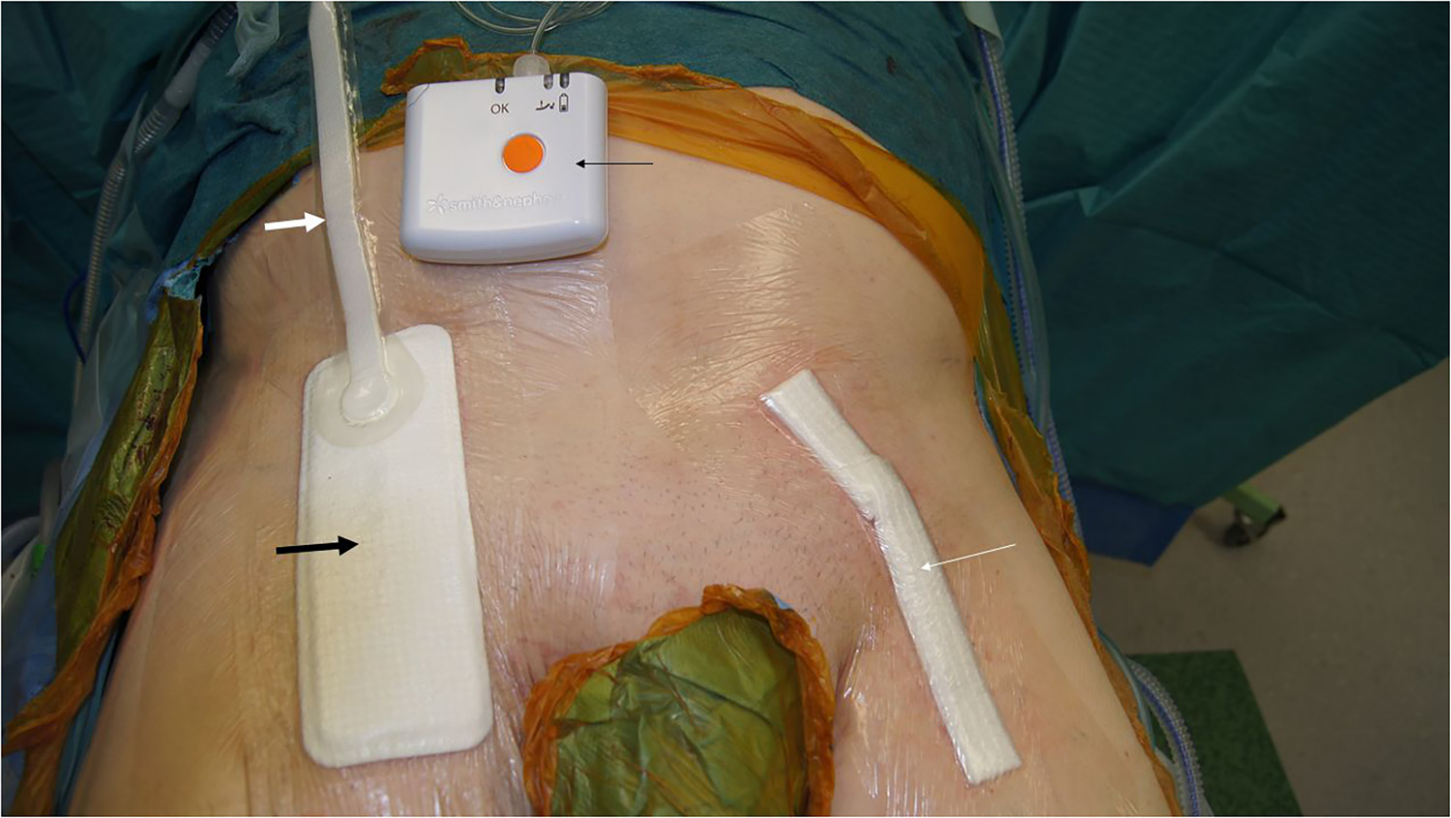
Patient undergoing bilateral thrombendarterectomy (TEA) of the common femoral artery in the randomized controlled trial (clinical trials.gov; NCT01913132). The PICO™ dressing system (Smith & Nephew, London, UK) was applied above the closed incision in the right groin. The pump (thin black arrow) provides continuous negative pressure to the PICO™ dressing pad (thick black arrow) through an interconnecting tube (thick white arrow). The standard dressing (Aquacel^®^, Convatec, Inc, and a transparent film dressing [Tegaderm™ Film, 3 M]) was applied above the closed incision in the left groin (thin white arrow). (Courtesy of F. Rezk, Unit of Vascular Surgery, Department of Surgery, Jönköping, Sweden).

Meta-analysis of randomized controlled trials (RCTs) in vascular surgical patients has shown a reduction of SSI after vascular surgery (3). The PICO™ dressing system (Figure 1) consists of a small pump creating negative pressure via an interconnecting tube. The dressing and pump are designed for single use and the battery drives the pump for 7 days. The aim of this study was to explore patients’ experiences of wearing the PICO™ dressing after vascular surgery of the lower limbs.

## Methods

A qualitative interview study was conducted. Participants were recruited from the ongoing multi-center RCT (Clinical Trials.gov; NCT01913132) comparing the PICO™ dressing system (Smith & Nephew) to standard dressings above closed incisions after elective arterial surgery in the lower limbs. In the case of bilateral arterial surgery with groin incisions, the right groin received the randomized dressing while the contralateral groin automatically received the comparator (4). Participants randomized to PICO™ between March 2020 and May 2021 were asked to participate before discharge from the hospital and were at the same time supplied with written information regarding the study. All patients that were approached for the study agreed to be interviewed.

All interviewed participants underwent lower limb revascularization, received PICO™ for 7 days after vascular surgery and were discharged with the PICO™ dressing. The interviews were conducted within 3 weeks after hospital discharge. Nine male and 6 female participants were interviewed. The median age was 77 years, range (65-84).

The study complies with the Standards for Reporting Qualitative Research (SRQR) (5). Telephone interviews with participants were conducted by the first author JN and recorded digitally due to the Covid-19 pandemic. The interviewer had been involved in the care of only one participant. Median interview length time was 10.53 min (range 6.07-16.14). Interviews were individual and semistructured following an interview guide developed by the research team.

The recorded interviews were transcribed and analyzed in accordance with Braun & Clarke thematic analysis (6). JN and ACA conducted the analysis, firstly one by one, and then together until a preliminary structure was reached. The transcriptions were read through several times, then initial units and codes were identified, and subthemes were formulated. The subthemes were further discussed, adjusted, and grouped into themes, related back to the aim. The themes and subthemes were reviewed and jointly discussed by all authors until consensus was reached, and the labels were decided. Citations from the interviews were marked in consecutive order (1-15), translated by the authors, and presented as examples of the results.

## Results

**Figure 2.**
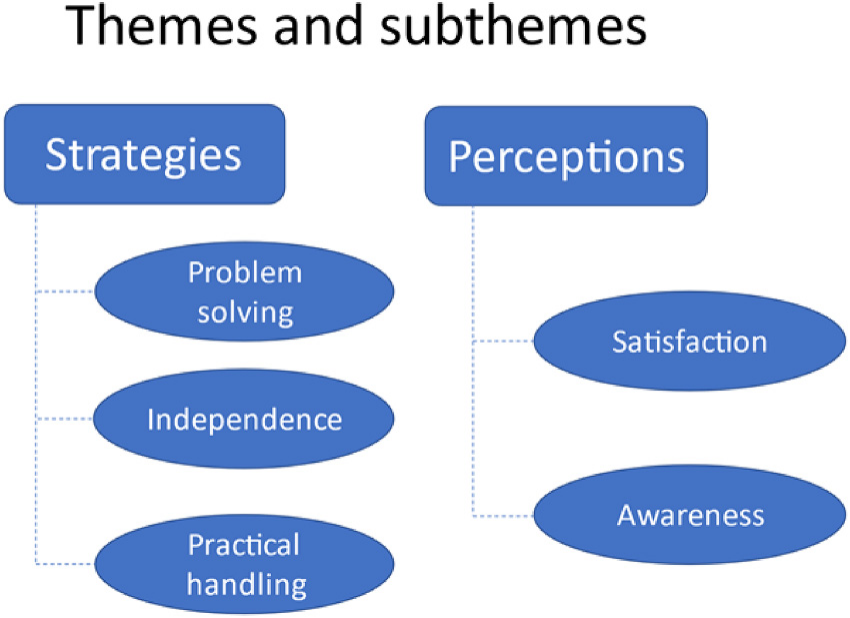
The 2 themes and 5 subthemes identified from the analysis.

The analysis resulted in 2 main themes, Strategies and Perceptions (Figure 2).

## Strategies

### Problem-Solving

In this subtheme, issues of irritation and fear of breaking the pump arose. None of the participants found it stressful when the pump malfunctioned or gave off an alarm. Some participants never found the reason for the beeps and light flashes and seemed to accept this without any further consideration. One participant took out the batteries and put them back in while another participant just put the pump off and then turned it on again. Participants handled problems that emerged from the PICO™ dressing without any aid from healthcare.

“I resolved the problems by myself every time. I didn't consider it to be any issue.” (Interview 6)

Many participants were unsure how much damage the pump could tolerate. This caused fear of breaking the pump and made the participants constantly aware of where the pump was positioned. No participant damaged the pump to the extent that it had to be replaced.

“I was scared of breaking it initially but after three falls I understood that it probably was reasonably durable.” (Interview 11)

### Independence

None of the participants considered the PICO™ dressing to interfere with their independence, whereas the initial pain after surgery limited them.

“I started to walk directly after the surgery. I haven’t been able to do that before because of poor arterial circulation in my leg so I experienced a revolution in the body” (Interview 3)

The patients felt safe and confident with the dressing at home. Some experienced a lack of information of how to handle the PICO™ dressing.

“I didn't get any information but I still felt confident coming home.” (Interview 5)

### Practical Handling

Some participants felt that their personal hygiene had deteriorated while wearing the PICO™ dressing. The participants who did shower with the dressing found it easy and uncomplicated.

“I was scared of showering, I didn’t want to do it so I used clean cloths instead. I was worried that the dressing would get wet underneath. It can spread bacteria in the wound.” (Interview 11)

“The dressing was so neat so taking a shower was no problem. They were water resistant” (Interview 7)

Some participants thought that the tube was too long and wished for a shorter tube. Some participants carried the pump in their pocket while others took use of the clip to secure it to the waist of their underwear or pants. The option to carry the pump in a bag with a shoulder sling was regarded as the safest option.

“It was a bit special. You had to carry the pump wherever you went. Initially, I had a bit of a problem with figuring out where to keep it.” (Interview 11)

## Perceptions

### Satisfaction

Many participants thought the PICO™ dressing had done a good job caring for their postoperative wound.

“I think it has been really good. I only had it for a week, and it has worked out really well.” (Interview 12)

One dissatisfied participant felt that the PICO™ dressing was mostly in the way but at the same time expressed that it was not a big burden to wear it. One participant complained of local pain. This participant had his skin incision closed with surgical staples and had the PICO™ dressing applied over the wound, which probably caused the PICO™ dressing to pull on the staples. Four participants who simultaneously got a PICO™ and standard dressing at opposite groins experienced the PICO™ dressing to be superior and more than one participant thought the incision looked cleaner.

“It has to be something because it healed up better than the other side.” (Interview 9)

### Awareness

Some participants felt they constantly had to make sure that the green light indicator was signaling or if there were any indication of malfunctioning. These participants considered that the information about the indicators at discharge was poor, if any. None of the participants needed to contact their healthcare professional due to pump malfunction.

“It wasn't very reliable. You had to keep an eye on it all the time” (Interview 6)

## Discussion

The present study found that most participants wore the PICO™ dressing with little impact on their daily life without needing to contact their healthcare professional. It was clear that the major event was the surgery and the changes in life that it implied.

Research on patients’ experiences of iNPWT for the treatment of closed wounds is scarce and there is a high demand for qualitative studies supplementing the quantitative RCTs (7).

Some participants expressed that it was challenging to always have control of the pump, not sleeping on it, and not understanding all sounds it made. The participants were most anxious to destroy the pump device by dropping it to the floor. A well-structured information at hospital discharge would have shorten the time needed to feel confident when wearing the dressing at home. The present study results might help the manufacturers to develop their patient information material. One patient experienced pain at the surgical site after wound closure by skin staples, probably due to PICO™ dressing-related pull on the staples. This finding needs further investigation.

Four participants who received PICO™ and standard dressing in opposite groins simultaneously preferred the PICO™ dressing. The aspect of the 7-day time limit for using the PICO™ dressing was also important. This gave the participants a defined time that helped them to cope with the dressing. No difference in experience between male and female participants was identified. Several other ultraportable devices for iNPWT are available on the market (8), which all merit to be investigated by similar qualitative interview studies.

Thematic analyses are sometimes seen as reflexive, producing meaningful knowledge (9). Therefore, the method suits this study, exploring how it is to be wearing the PICO™ dressing.

## Limitations

Although the interviews were rather short, the data material was quite rich and substantial, and saturation was achieved in the material. The same interviewer conducted all the interviews and the transcription. Participants were enrolled from 4 centers, located in different Swedish regions, increasing trustworthiness, credibility, and transferability of the data (10).

## Conclusion

This study implicates that the PICO™ dressing can be used with little discomfort to most patients after elective vascular surgery.
